# Predictive value of D-dimer and analysis of risk factors in pregnant women with suspected pulmonary embolism after cesarean section

**DOI:** 10.1186/s12890-021-01757-3

**Published:** 2021-12-01

**Authors:** Limin Zhang, Yunqiu Chen, Wenjuan Liu, Xinzhuo Wang, Shuang Zhang, Wenyan Zhang, Shuai Zhao, Miaomiao Zhang, Su Zhang, Guangyu Jiao

**Affiliations:** Department of Pulmonary and Critical Care Medicine, Shengjing Hospital of China Medical University, Sanhao Street, Shenyang, 110004 Liaoning China

**Keywords:** Acute pulmonary embolism, D-dimer, Cesarean section, Diagnosis, Risk factors

## Abstract

**Background:**

Acute pulmonary embolism (PE) is one of the leading causes of maternal mortality, and cesarean section is an established independent risk factor for PE. The diagnostic utility of D-dimer for PE in non-pregnant women has been well-established, but its role in women with suspected PE after cesarean section is unclear. Furthermore, the optimal threshold level in this patient population is unknown. Traditional D-dimer levels have low diagnostic specificity, resulting in many pregnant women being exposed to potentially harmful radiation despite negative diagnostic imaging results. This research aimed to optimize the clinical threshold for D-dimer to improve specificity while ensuring high sensitivity and to identify risk factors for PE after cesarean section.

**Methods:**

This retrospective study of 289 women who underwent diagnostic imaging (ventilation/perfusion [V/Q] or computed tomographic pulmonary angiography [CTPA]) for suspected acute PE after cesarean delivery from 2010 to 2021 was conducted. Clinical data and laboratory indicators within 24 h postpartum including D-dimer levels were collected for analyses.

**Results:**

The final analysis included 125 patients, among whom 33 were diagnosed with acute PE (incidence of 11.42%, 95% confidence interval 7.7–15.1). The receiver operating characteristic curve analysis suggested that a D-dimer cut-off value of 800 ng/mL had specificity of 25.26% and sensitivity of 100% for detecting PE. The cut-off value was adjusted to 1000 ng/mL with a specificity of 34.74% and a sensitivity of 96.67%. Using a D-dimer cut-off value of 800 ng/mL (instead of the conventional value of 500 ng/mL) increased the number of patients excluded from suspected PE from 9.6 to 18.4% without additional false-negative results. Of note, a history of known thrombophilia was significantly more common in patients with PE than in those without (*P* < 0.05). No other independent risk factors were noted in our study.

**Conclusions:**

The D-dimer cut-off value of 800 ng/mL ensures high sensitivity and increases specificity compared to the conventional threshold of 500 ng/mL. Utilizing this higher threshold can reduce the number of unnecessary CT and subsequently unnecessary radiation exposure, in women after cesarean delivery. Prospective studies should also be conducted to verify these results.

**Supplementary Information:**

The online version contains supplementary material available at 10.1186/s12890-021-01757-3.

## Background

Acute pulmonary embolism (PE) remains one of the leading direct causes of death in pregnancy and postpartum [1–3]. In the United States, PE contributes to 9.2% of pregnancy-related deaths, or 1.13 deaths per 100,000 births [4]. Pregnant women have a much higher risk of PE compared to non-pregnant women of the same age, and this risk increases during pregnancy and peaks after delivery [5]. PE-related morbidity and mortality is especially high after cesarean delivery. The cumulative incidence of PE after cesarean delivery was 4 times higher than after vaginal delivery, with the majority (66.7%) occurring within 2 weeks postpartum [6].

The clinical symptoms of PE overlap significantly with those caused by physiological changes during pregnancy, such as tachycardia, leg swelling, and dyspnea, which presents a diagnostic challenge [7]. Pregnant women with suspected PE frequently present to emergency departments and obstetric units. Currently, the diagnosis of suspected PE in pregnant women mainly relies on chest imaging, namely computed tomographic pulmonary angiography (CTPA) and/or lung ventilation/perfusion (V/Q) scans. However, the diagnostic yield of these imaging modalities reported in a recent North American diagnostic study was as low as 5%, suggesting that many pregnant women were subjected to potentially harmful radiation despite not having a PE [8, 9]. Further, some studies have suggested that CTPA and pulmonary angiography can increase a woman's risk of breast cancer [10–12]. Given that pregnant or lactating women are already estimated to be at higher risk due to perinatal breast tissue hyperplasia, albeit to an unknown extent, this is an important consideration [13, 14].

Among the screening tools for PE, multiple studies have demonstrated the reliability of the D-dimer assay which has high sensitivity and moderate specificity in non-pregnant women [15]. The D-dimer assay combined with adequate pretrial probability estimation can promote the safe discharge of patients with suspected PE and reduce unnecessary investigations or anticoagulation [16]. D-dimer is a specific degradation product of cross-linked fibrin, and elevated D-dimer level is a manifestation of fibrinolytic hyperactivity, which has important diagnostic value for thrombotic diseases. However, plasma D-dimer level is affected by various factors and can be increased in malignancy, infection, postoperatively, and in special physiological conditions such as advanced age or pregnancy [17–19]. Therefore, the effectiveness of D-dimer as a screening tool for PE may decrease in certain populations. As previously mentioned, the concentration of D-dimer gradually increases during pregnancy and reaches a peak on the first day after delivery [20]. Based on the physiological status of pregnant women, the current recommended reference range of plasma D-dimer levels (≤ 500 ng/mL) for venous thromboembolism (VTE) in normal women does not apply to pregnant and perinatal women, which increases the false positive rate and leads to unnecessary imaging and anticoagulation treatments. Owing to the lack of strong evidence to validate the diagnostic algorithm, there is no consensus in the international guidelines for the diagnosis of PE in pregnancy. The guidelines of the American Thoracic Society [21] and the Royal College of Obstetricians and Gynecologists [22] recommend that all pregnant and postpartum women with suspected PE should undergo diagnostic imaging investigations, while the guidelines of the European Society of Cardiology [23] suggest that D-dimer may play a role in screening pregnant and postpartum women with suspected PE. Several studies have attempted to demonstrate the predictive value of the D-dimer test by raising the cut-off value for pregnancy-related VTE or finding a higher D-dimer reference range [24–29]. However, the role of D-dimer has not been verified in women after cesarean section, and there is currently a lack of clinical studies to evaluate the diagnostic value of D-dimer in maternal PE after cesarean section.

Therefore, we conducted a retrospective study to establish a higher D-dimer diagnostic threshold for women with PE after cesarean section. Furthermore, we explored the risk factors for PE after cesarean section in women with suspected PE.

## Methods

### Patients

Shengjing Hospital of China Medical University has one of the largest obstetrics and gynecology department in China, with 736 beds. Every year, 10,000 pregnant women are discharged from hospital, of whom more than 60% are considered high-risk pregnancies, and the number of deliveries reaches 11,000.

**Fig. 1:**
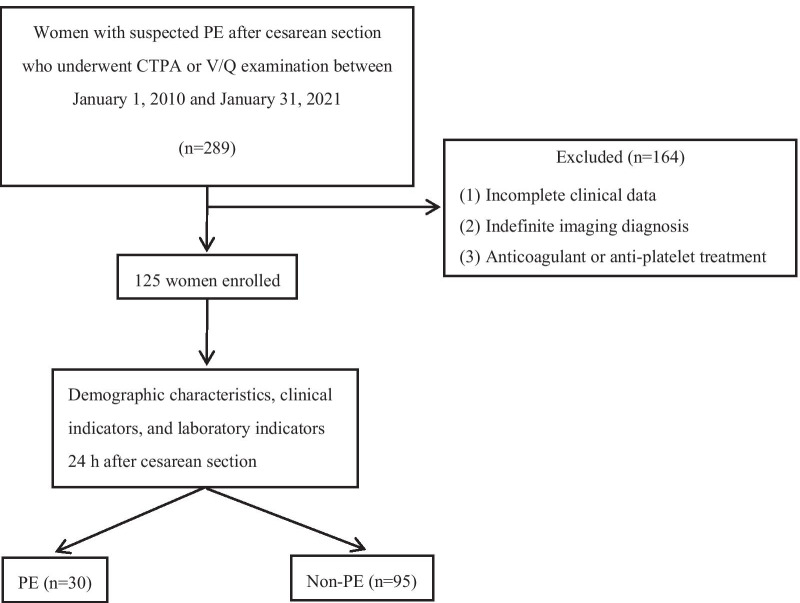
Flow chart of participant selection

A retrospective analysis was conducted on women with suspected PE after cesarean section in the obstetrics and emergency department of our hospital from January 2010 to January 2021, who underwent imaging examinations with V/Q scan, CTPA, or pulmonary angiography (Fig. 1). PE was diagnosed based on the PIOPED criteria [30, 31]: high-probability V/Q scan without previous history of PE, i.e., ≥ 2 segmental perfusion defects (V/Q mismatch); or positive CTPA scan or pulmonary angiography.

Patients with one or more of the following criteria were excluded from the study: (1) indefinite imaging diagnosis; (2) anticoagulant or anti-platelet treatment; and (3) missing data.

D-dimer concentration was determined using the ACL-TOP700 Coagulometer (Instrumentation Laboratory, America) by an immunoturbidimetric assay that has the same high sensitivity as the ELISA assay (the normal reference range for non-pregnant adults is below 500 ng/mL).

The shock index (SI) was calculated as heart rate (HR)/systolic blood pressure.

### Data analysis

SPSS version 22.0 software (IBM Corporation, New York) was used for data analysis. Measurement data conforming to normal distribution and homogeneity of variance are expressed as mean ± standard deviation (x ± s), and independent-sample T test was used for comparison between groups. Measurement data that did not conform to normal distribution or homogeneity of variance were represented by median and quad ranges, and comparisons between groups were performed by Mann–Whitney U test. Differences in categorical variables were compared by chi-squared test or Fisher's exact probability method. Risk factors of PE were estimated by logistic multivariate regression analysis model, and the odds ratio (OR) and 95% confidence interval (CI) were calculated. The ROC curve was analyzed to obtain 95% CIs for sensitivity and specificity, positive (PVV) and negative predictive values (NPV). Statistical significance was set at *P* < 0.05.

## Results

### Patient characteristics

From 2010 to 2021, a total of 289 women underwent V/Q scans or CTPAs as part of the assessment of suspected PE after cesarean section in our hospital. No patients underwent pulmonary angiography. Among them, 11 women (3.81%) did not have radiological evidence of PE but this could not be definitely ruled out. Thirty-three women were finally diagnosed with PE, with an incidence of 11.42% (95% CI 7.7–15.1). Thirteen women received aspirin or low-molecular-weight heparin (LMWH) prior to the D-dimer test. A total of 125 people were included in the study based on inclusion and exclusion criteria. There were 30 patients in the PE group (mean age, 31.27 ± 5.29 years; range 22–43 years) and 95 patients in the non-PE group (mean age, 31.62 ± 5.07 years; range 21–43 years). There was no statistical difference in age between the two groups (*P* > 0.05). The main clinical symptoms of the two groups were dyspnea (36 cases), chest distress and tachypnea (37 cases), hypoxemia (26 cases), chest pain (15 cases), hemoptysis (5 cases), cardiac and respiratory arrest (2 cases), syncope (3 cases) and sudden death (1 case). Symptoms of suspected PE mostly developed on the first day after cesarean section (62.07%), and the median time of onset was 1 day after delivery (range 1–7 days).

### General data analysis

We compared clinical characteristics and laboratory indicators within 24 h after cesarean section between the PE and non-PE groups (Table 1). There were no significant differences in maternal age, body mass index, fetal length, gestational weeks, neonatal weight, neonatal Apgar score, modified Wells score, mean arterial pressure, fibrinogen, platelet distribution width, red blood cell distribution width, creatine kinase (CK) and CK-MB between the two groups (*P* > 0.05). There were significant differences in SI and D-dimer level between the two groups (*P* < 0.05). The mean D-dimer level at 24 h postpartum in the PE group was significantly higher than that in the non-PE group (13.7 mg/L vs 2.35 mg/L, *P* = 0.000).

**Table 1:** Comparison of general characteristics between PE and non-PE groups after cesarean section (x ± s or median [P25, P75])

	PE N = 30	Non-PE n = 95	Coefficient	*P* value
Age (years)	31.27 ± 5.29	31.62 ± 5.07	− 0.330	0.752^a^
BMI (kg/m^2^)	27.99 ± 4.76	26.54 ± 3.77	0.982	0.324^a^
Gestational age at delivery (weeks)	35.69 ± 2.66	34.48 ± 4.10	1.228	0.073^a^
Neonatal birth weight (g)	2750.00 ± 744.81	2264.82 ± 859.06	2.060	0.637^a^
Neonatal body length (cm)	47.18 ± 4.53	44.22 ± 5.60	− 1.971	0.053^a^
Mean arterial pressure (mmHg)	93.31 ± 19.55	101.85 ± 16.10	− 2.370	0.443^a^
Shock index	0.87 [0.58, 1.12]	0.70 [0.62, 0.80]	− 1.931	0.042^b^
Apgar score at 1 min	10.00 [4.00, 10.00]	9.00 [7.00, 10.00]	− 0.053	0.957^b^
Apgar score at 5 min	10.00 [9.00, 10.00]	10.00 [10.00, 10.00]	− 0.276	0.782^b^
Modified Wells score	3.0 [1.5, 6.0]	3.0 [1.5, 4.5]	− 1.271	0.204^b^
Laboratory test results within postpartum 24 h				
D-dimer (mg/L)	3.00 [1.01, 6.91]	1.24 [7.54, 2.38]	− 4.659	0.000^b^
Fibrinogen (g/L)	3.50 [2.70, 4.53]	3.80 [3.20, 4.30]	− 0.900	0.368^b^
PDW (%)	16.95 [15.30, 17.60]	17.40 [16.20, 18.20]	− 1.491	0.136^b^
RDW (%)	14.50 [13.13, 17.50]	14.50 [13.50, 16.80]	− 0.286	0.775^b^
CK (u/L)	130.50 [58.25, 256.75]	128.50 [84.90, 221.25]	− 0.225	0.822^b^
CK-MB (u/L)	22.00 [13.00, 72.75]	27.00 [18.00, 44.40]	− 0.384	0.701^b^

BMI, body mass index; PDW, platelet distribution width; RDW, red cell distribution width; CK, creatine kinase; CK-MB, creatine kinase myocardial band

^a^Evaluated using the t test

^b^Evaluated using the Mann–Whitney U test

### Determining the cut-off value of D-dimer

**Fig. 2:**
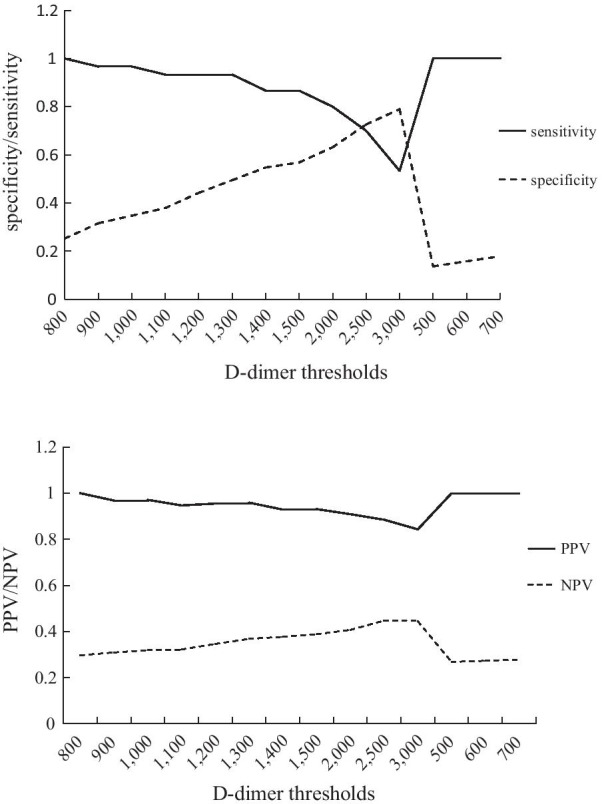
Sensitivity, specificity, PPV, and NPV of the evaluated D-dimer thresholds in the diagnosis of acute PE after cesarean section

In this study, the D-dimer level in most women (113, 90.4%) after cesarean section exceeded the upper limit of the normal reference value (500 ng/mL). The ROC curve analysis showed that the area under the ROC curve (AUC) of D-dimer was greater than 0.7 (Additional file 1: Analysis of ROC), and there was a statistical correlation with PE (*P* = 0.000), indicating significant diagnostic value for PE detection. Figure 2 illustrates the sensitivity, specificity, PPV, and NPV at different D-dimer cut-off values. When the D-dimer cut-off value was set at 500 ng/mL, the sensitivity and NPV were 100.00% with a specificity of 13.68% and a PPV of 26.80%. When the cut-off value was adjusted to 800 ng/mL, the specificity increased to 25.26% and PPV increased to 29.70% while maintaining a sensitivity and NPV of 100.00%. When the cut-off value was adjusted to 1000 ng/mL, the specificity increased to 34.74% and PPV increased to 31.90%, while the sensitivity and NPV decreased (96.67% and 97.10%, respectively). Internal cross-validation confirmed the reliability of the 800 ng/mL cut-off value with sensitivity and NPV of 100.00% (Additional file 2: Internal cross validation).

### Risk factor analysis

We also analyzed the risk factors for PE in women after cesarean section (Table 2). Demographic data (advanced age, obesity, number of parities and delivery, macrosomia), pregnancy characteristics (premature birth, placenta previa, premature rupture of membranes, placental abruption, uterine fibroids), medical comorbidities (gestational hypertension, gestational diabetes mellitus, chronic heart disease, systemic lupus erythematosus, history of thrombosis), and delivery complications (general anesthesia, emergency cesarean section, postpartum hemorrhage, blood transfusion, postpartum infection) were not significantly associated with PE. The rate of known thrombophilia in women with PE was significantly higher than that in women without PE (33.33% vs 12.63%, *P* = 0.014).

**Table 2:** Risk factors for PE in women after cesarean section

Risk factors	PE (%) (n = 30)	Non-PE (%) (n = 95)	χ^2^ value	*P* value
Demographic data				
Age (> 35 years)	7 (23.33)	22 (23.16)	0.000	0.984
Obesity (BMI ≥ 30 kg/m^2^)	8 (26.67)	16 (17.78)	1.419	0.234
Twins or multiplets	3 (10.00)	18 (18.95)	1.306	0.253
Parity > 1	12 (40.00)	34 (35.79)	0.174	0.677
Gravidity > 1	17 (56.67)	65 (68.42)	1.396	0.237
Macrosomia (birthweight ≥ 4000 g)	0 (0.00)	2 (2.11)	–	1.000^c^
Pregnancy characteristics				
Premature birth	19 (63.33)	69 (72.63)	0.946	0.331
Placenta previa	2 (6.67)	6 (6.32)	–	1.000^c^
Premature rupture of membranes	5 (16.67)	14 (14.74)	–	0.776^c^
Placental abruption	7 (23.33)	13 (13.68)	–	0.254^c^
Uterine fibroids	3 (10.00)	8 (8.42)	–	0.724^c^
Medical comorbidities				
Gestational diabetes mellitus	7 (23.33)	23 (24.21)	0.010	0.922
Pregnancy-related hypertension	12 (40.00)	49 (51.58)	1.223	0.269
Chronic heart disease	4 (13.33)	14 (14.74)	–	1.000^c^
Systemic lupus erythematosus	0 (0.00)	1 (1.05)	–	1.000^c^
Known thrombophilia	10 (33.33)	12 (12.63)	6.738	0.014
History of thrombosis	1 (3.33)	0 (0.00)	–	0.240^c^
Delivery complications				
General anesthesia	32 (33.68)	12 (40.00)	0.399	0.528
Emergency CS	23 (76.67)	63 (66.32)	1.138	0.286
Postpartum hemorrhage	6 (20.00)	11 (11.58)	–	0.238^c^
Postpartum blood transfusion	7 (23.33)	20 (21.74)	0.070	0.791
Intraoperative blood transfusion	2 (6.67)	3 (3.16)	–	0.593
Postpartum infection	3 (10.00)	4 (4.21)	–	0.357

The logistic risk model was established by the forward stepwise regression method to identify the independent risk factors for PE after cesarean section. D-dimer was an independent predictor of PE after cesarean section (*P* = 0.010) (Table 3).

**Table 3:** Logistic regression of PE in women after cesarean section

Risk factors	B	SE	Wald	*P* value	OR	95% CI	
D-dimer	0.177	0.069	6.655	0.010	1.194	1.043	1.366
SI	0.856	0.883	0.940	0.332	2.354	0.417	13.286
Constant	− 2.537	0.738	11.817	0.001	0.079		

## Discussion

The incidence of PE in pregnant women is approximately 10 times that of non-pregnant women of the same age [32]. Postpartum is a high-risk period for the occurrence of VTE, especially after cesarean section. Numerous studies have shown that cesarean section is an independent risk factor for PE [33–35]. Since most signs and symptoms of perinatal PE are non-specific, a higher suspicion index is necessary to ensure timely accurate diagnosis and initiation of appropriate anticoagulation treatment. Our study revealed that the incidence of PE in women with suspected PE after cesarean section undergoing CTPA or V/Q scans was 11.42% (95% CI 7.7–15.1). Previous reports have shown that the incidence of PE in pregnant women with suspected PE is 5% or less, compared with 15% to 20% among non-pregnant women [36, 37]. Therefore, the incidence of suspected PE in our study population was higher than that previously reported in pregnant women. This may be because cesarean section is the vital independent risk factor for PE. Since the use of aspirin or LMWH may affect D-dimer results, we excluded patients with a history of anticoagulation from our study. In fact, only 13 patients received aspirin or LMWH in this study, which likely had minimal effect on the incidence of PE.

D-dimer has a high NPV for VTE, but D-dimer levels continue to increase throughout pregnancy and are higher than the normal reference values for most healthy pregnant and postpartum women [38]. In a prospective study [39], 84% of pregnant women had normal D-dimers in the first trimester, 33% in the second trimester, and only 1% in the third trimester, which suggests that the conventional D-dimer threshold has no practical diagnostic value for maternal PE. At present, there are controversies about the diagnostic threshold value of D-dimer for pregnant and postpartum women to exclude acute PE, and the optimal threshold value is still inconclusive. Many studies support D-dimer assays with higher thresholds that increase D-dimer specificity while maintaining high sensitivity. A prospective study showed that PE was safely ruled out by the pregnancy-adapted YEARS algorithm across all trimesters of pregnancy [40]. In the algorithm, PE was ruled out if patients had a D-dimer level less than 1000 ng/mL with none of the following three criteria: clinical signs of DVT, hemoptysis, and PE as the most likely diagnosis; or if the D-dimer level was less than 500 ng/mL with evidence of one or more of the three criteria. However, the D-dimer threshold value of the pregnancy-adapted YEARS algorithm for PE exclusion in women after cesarean section is still undefined. Further, no study has evaluated the D-dimer cutoff value for PE diagnosis 24 h after cesarean section. In our study, we collected and analyzed the data of women with suspected PE after cesarean section in our hospital over the past 11 years. We found that most women with suspected PE had similar clinical manifestations of PE on the first day after delivery (accounting for 62.07%), and D-dimer detection within 24 h postpartum had a significant diagnostic value for detecting PE after cesarean section. The high sensitivity and safety of the D-dimer threshold in the Pregnancy-Adapted YEARS Algorithm in the diagnosis of maternal PE after cesarean section was further verified in our study. Our study attempted to provide a higher D-dimer cut-off value to exclude acute PE in women after the cesarean section. A D-dimer cut-off value of 800 ng/mL (instead of the standard 500 ng/mL) increased the percentage of women in whom PE was ruled out from 9.6 to 18.4%, with no additional false-negative results. Therefore, we believe that D-dimer detection within 24 h after cesarean section in women with suspected PE is necessary. Together with clinician experience, abnormally elevated D-dimer levels can help clinical decision-making regarding the indications for and treatment duration with prophylactic LMWH. Based on our results, we suggest that when the D-dimer level is higher than 1000 ng/mL in women with suspected PE after cesarean section, anticoagulation should be initiated and patients should undergo diagnostic imaging. Conversely, PE can basically be ruled out when the D-dimer level is below 800 ng/mL.

Previous studies have suggested that various complications of pregnancy and childbirth, age over 35 years, obesity, heart disease, and other chronic diseases are important risk factors for maternal PE [41–45]. One retrospective study in Taiwanese women undergoing cesarean section found that chronic heart disease, systemic lupus erythematosus, postpartum hemorrhage, blood transfusion, and postpartum infection were strong perioperative risk factors for postpartum PE [46]. However, we did not find differences in these factors between women with and without PE in our study. The reason for the different results may be owing to differences in the study population and the fact that cesarean section was the strongest predictor of PE. Further, we found that the proportion of patients with known thrombophilia was significantly higher in the PE group than in the non-PE group. Since thrombophilia is a well-established risk factor for thrombosis, this may have influenced our results [47]. We also found that the modified Wells score has no significant predictive value for PE after cesarean section; this may be because almost all women have postoperative risk factors for VTE after cesarean section and non-specific clinical manifestations that mimic VTE, such as lower limb edema. Some studies have indicated that the SI has a predictive value in the diagnosis of PE. A multicenter study has shown that SI is highly sensitive in identifying a subgroup of patients with a low risk of death and can accelerate the diagnosis of patients with suspected acute PE [48, 49]. Although we found significant differences in SI between the two groups, SI did not correlate with PE. Therefore, SI has no practical diagnostic significance for PE after cesarean section in our study, which is consistent with a previous study [27]. Further, pregnancy is characterized by physiological changes in the cardiovascular system, including variations in HR and blood pressure, and these can be further influenced by surgery including cesarean section. Since the SI is the ratio of HR to systolic blood pressure, it may be influenced by normal changes in pregnancy or mode of delivery; therefore, the role of SI in maternal PE remains uncertain. Reason for the different results may be related to the different study populations and collection times. Further large-scale and prospective studies are needed to evaluate the predictive ability of the SI in the diagnosis of PE after cesarean section.

Few studies have analyzed the risk factors for PE after cesarean section. We found that many of the traditional risk factors for PE have little diagnostic value in the post-cesarean population and may be misleading in the diagnostic evaluation of women with suspected PE after cesarean section. Therefore, clinicians need to consider the unique risk factors for PE after cesarean section to ensure appropriate diagnostic work-up and treatment.

Our study has some limitations. First, some women with suspected PE did not undergo CTPA or V/Q scans owing to patient refusal, which led to selection bias. Second, although this retrospective analysis included data obtained over the last 11 years, the sample size was relatively small; nevertheless, this was consistent with the incidence of maternal PE. The accuracy of D-dimer for the diagnosis of PE after cesarean section needs to be further verified by expanding the sample size. Third, we found no significant difference in age between the two groups (*P* = 0.986); however, we could not perform stratified analyses according to age because of the small number of patients included. Further studies with larger sample sizes are needed to explore the differences in D-dimer levels across different age groups. We will continue to collect data and hope to establish more accurate D-dimer cut-off values by expanding the sample size. We expect to refine the threshold value at different timepoints after cesarean section and identify more meaningful risk factors for PE in this population.

## Conclusions

The incidence of PE in women after cesarean section is significantly increased, and the diagnostic sensitivity of the traditional D-dimer cut-off value is distinctly reduced in this patient population. Therefore, it is essential to identify a higher D-dimer cut-off value, which improves diagnostic specificity while maintaining high sensitivity, thereby allowing for a safe reduction in the number of CT scans performed in women after cesarean section. We did not find other independent risk factors for suspected PE after cesarean section. Although our study supports higher D-dimer thresholds to exclude acute PE in women after cesarean section, prospective studies and external validation should be conducted to verify our results.

## Supplementary Information

**Additional file 1.** Analysis of ROC.

**Additional file 2.** Internal cross validation.

**Additional file 3.** Ethical approval documents.

## Data Availability

The datasets used and/or analysed during the current study are available from the corresponding author on reasonable request.

## Abbreviations

AUC: Area under the curve
CTPA: Computed tomographic pulmonary angiography
DVT: Deep venous thrombosis
NPV: Negative predictive value
PE: Pulmonary embolism
PPV: Positive predictive value
ROC: Receiver operating characteristic
SI: Shock index
V/Q: Lung ventilation/perfusion
VTE: Venous thromboembolism
