# Supplementary material for: Predictive value of D-dimer and analysis of risk factors in pregnant women with suspected pulmonary embolism after cesarean section
Source: BMC Pulm Med. 2021 Dec 1;21:391. doi: 10.1186/s12890-021-01757-3 (PMC8638256; doi:10.1186/s12890-021-01757-3)

**Fig. S1** Receiver operating characteristic (ROC) curve for D-dimer, shock index and PDW within 24h of cesarean section.

AUC (ROC of D-dimer): 0.783 (95% CI: 0.696-0.869, P =0.000); AUC (ROC of SI): 0.625 (95% CI: 0.488-0.762, P =0.042); AUC (ROC of FIB): 0.445 (95% CI: 0.309-0.580, P =0.368)


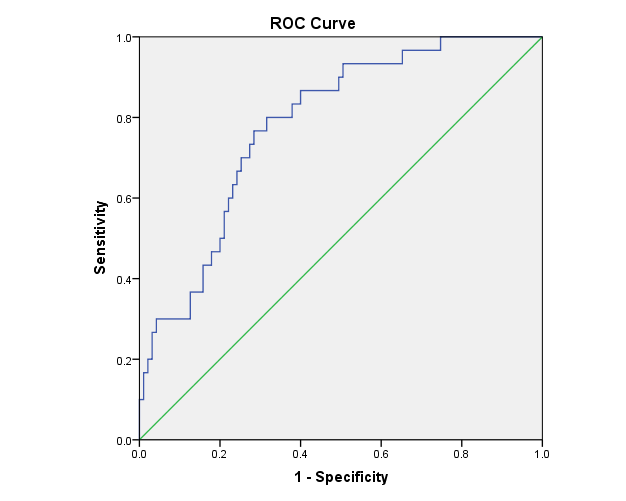

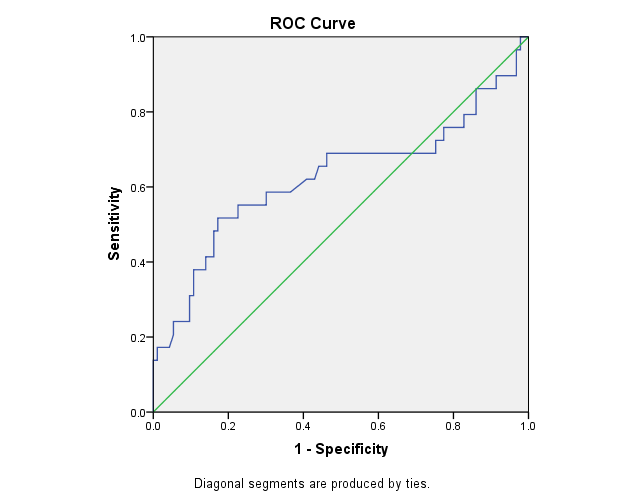

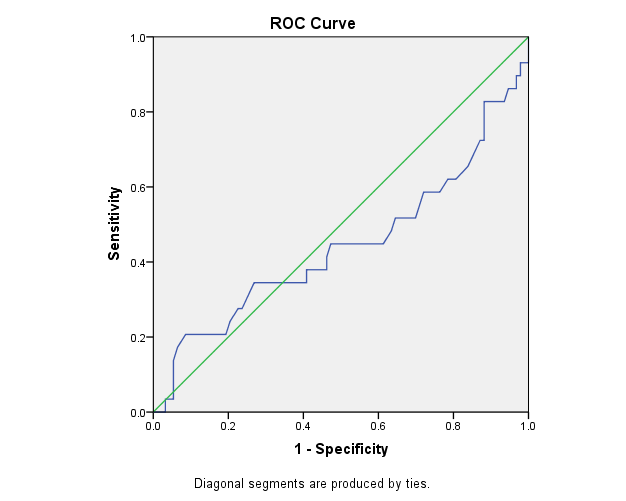

Supplement: Supplementary file 1 — Additional file 1. Analysis of ROC. [file 12890_2021_1757_MOESM1_ESM.docx]
