# Supplementary material for: Predictive value of D-dimer and analysis of risk factors in pregnant women with suspected pulmonary embolism after cesarean section
Source: BMC Pulm Med. 2021 Dec 1;21:391. doi: 10.1186/s12890-021-01757-3 (PMC8638256; doi:10.1186/s12890-021-01757-3)
Supplement: Supplementary file 2 — Additional file 2. Internal cross validation. [file 12890_2021_1757_MOESM2_ESM.pdf]

Internal cross validation 1

DD(ug/L)	Sensitivity	1 - Specificity
309	1	1
318.5	1	0.987
339.5	1	0.974
366	1	0.961
393.5	1	0.947
408	1	0.934
428.5	1	0.921
453.5	1	0.908
479	1	0.895
500	1	0.882
501.5	1	0.868
560.5	1	0.855
619.5	1	0.842
662	1	0.829
722.5	1	0.816
744	1	0.789
748	1	0.776
784	1	0.763
856	0.958	0.763
924	0.958	0.75
977.5	0.958	0.737
1004.5	0.958	0.724
1028.5	0.917	0.724
1065	0.917	0.711
1103.5	0.917	0.697
1128	0.917	0.684
1140.5	0.917	0.671
1166	0.917	0.658
1185.5	0.917	0.645
1190	0.917	0.632
1203	0.917	0.618
1223	0.917	0.605
1239.5	0.917	0.592
1255.5	0.917	0.579
1282.5	0.917	0.566
1329.5	0.917	0.553
1368	0.917	0.539
1388.5	0.917	0.526
1399	0.917	0.513
1406	0.917	0.5
1463.5	0.917	0.487
1516.5	0.917	0.474

1542	0.917	0.461
1648	0.917	0.447
1797.5	0.917	0.434
1906	0.917	0.421
1959.5	0.917	0.408
2070.5	0.875	0.408
2175.5	0.875	0.395
2194	0.875	0.382
2276	0.875	0.368
2355.5	0.875	0.355
2375	0.833	0.355
2387	0.833	0.342
2397	0.833	0.329
2438.5	0.833	0.316
2486	0.792	0.316
2517	0.75	0.316
2583	0.75	0.303
2655.5	0.75	0.289
2735.5	0.708	0.289
2808	0.708	0.276
2827	0.708	0.263
2842.5	0.667	0.263
2906	0.667	0.25
2980.5	0.667	0.237
3027	0.625	0.237
3070.5	0.583	0.237
3091.5	0.583	0.224
3207.5	0.542	0.224
3379	0.542	0.211
3498.5	0.5	0.211
3599	0.5	0.197
3654	0.5	0.184
3796.5	0.458	0.184
4020	0.417	0.184
4328.5	0.417	0.171
4581.5	0.417	0.158
4628.5	0.417	0.145
4939.5	0.375	0.145
5263	0.333	0.145
5332.5	0.333	0.132
5571.5	0.333	0.118
5966	0.333	0.105
6194	0.333	0.092
6360.5	0.333	0.079

6694	0.333	0.066
6897	0.333	0.053
7437.5	0.292	0.053
7991	0.292	0.039
8207	0.25	0.039
8997	0.208	0.039
9751	0.208	0.026
11105	0.167	0.026
13917.5	0.167	0.013
16600	0.125	0.013
18137.5	0.083	0.013
30484.5	0.083	0
128719	0.042	0
215071	0	0

Internal cross validation 2

DD(ug/L)	Sensitivity	1 - Specificity
8.8	1	1
159.9	1	0.987
318.5	1	0.974
339.5	1	0.961
366	1	0.947
391.5	1	0.934
425.5	1	0.921
453.5	1	0.908
459.5	1	0.895
481	1	0.882
560.5	1	0.868
619.5	1	0.855
662	1	0.842
714.5	1	0.829
733	1	0.816
744	1	0.803
748	1	0.789
759	1	0.776
772	1	0.763
797	1	0.75
821.5	0.958	0.75
826.5	0.958	0.737
843	0.958	0.724
858.5	0.958	0.711
876.5	0.958	0.697
903	0.958	0.684
934	0.958	0.671

965.5	0.958	0.658
988	0.958	0.645
1004.5	0.958	0.632
1028.5	0.917	0.632
1086.5	0.917	0.618
1128	0.917	0.605
1140.5	0.917	0.592
1166	0.917	0.579
1186.5	0.917	0.566
1201.5	0.917	0.553
1213.5	0.917	0.539
1223	0.917	0.526
1239.5	0.917	0.513
1255.5	0.917	0.5
1282.5	0.917	0.487
1320.5	0.917	0.474
1348	0.875	0.474
1357.5	0.875	0.461
1373	0.833	0.461
1393	0.833	0.447
1399	0.833	0.434
1406	0.833	0.421
1463.5	0.833	0.408
1516.5	0.833	0.395
1542	0.833	0.382
1648	0.833	0.368
1759.5	0.833	0.355
1827	0.792	0.355
2017	0.792	0.342
2192.5	0.792	0.329
2281	0.792	0.316
2354.5	0.792	0.303
2364	0.792	0.289
2375	0.75	0.289
2387	0.75	0.276
2397	0.75	0.263
2438.5	0.75	0.25
2476.5	0.708	0.25
2490.5	0.708	0.237
2517	0.667	0.237
2583	0.667	0.224
2655.5	0.667	0.211
2742	0.625	0.211
2814.5	0.583	0.211

2827	0.583	0.197
2847	0.542	0.197
2910.5	0.5	0.197
2980.5	0.5	0.184
3027	0.458	0.184
3071	0.417	0.184
3103.5	0.375	0.184
3219	0.375	0.171
3379	0.375	0.158
3535.5	0.333	0.158
3778.5	0.333	0.145
4229.5	0.292	0.145
4581.5	0.292	0.132
4936	0.292	0.118
5263	0.25	0.118
5332.5	0.25	0.105
5780.5	0.25	0.092
6194	0.25	0.079
6427	0.25	0.066
6777.5	0.25	0.053
7437.5	0.208	0.053
7991	0.208	0.039
8207	0.167	0.039
8997	0.125	0.039
9751	0.125	0.026
11105	0.083	0.026
13917.5	0.083	0.013
17063.5	0.042	0.013
25011.5	0.042	0

Internal cross validation 3

DD(ug/L)	Sensitivity	1 - Specificity
8.8	1	1
168.4	1	0.987
339.5	1	0.974
377.5	1	0.961
405	1	0.947
408	1	0.934
428.5	1	0.921
454	1	0.908
479.5	1	0.895
500	1	0.882
501.5	1	0.868
560.5	1	0.855

619.5	1	0.842
662	1	0.829
714.5	1	0.816
733	1	0.803
755	1	0.776
772	1	0.763
797	1	0.75
821.5	0.958	0.75
826.5	0.958	0.737
843	0.958	0.724
858.5	0.958	0.711
876.5	0.958	0.697
903	0.958	0.684
934	0.958	0.671
965.5	0.958	0.658
992.5	0.958	0.645
1028.5	0.917	0.645
1065	0.917	0.632
1132	0.917	0.618
1185.5	0.917	0.605
1190	0.917	0.592
1201.5	0.917	0.579
1221.5	0.917	0.566
1239.5	0.917	0.553
1255.5	0.917	0.539
1301	0.917	0.526
1348	0.875	0.526
1357.5	0.875	0.513
1368.5	0.833	0.513
1383.5	0.833	0.5
1394	0.833	0.487
1457.5	0.833	0.474
1516.5	0.833	0.461
1542	0.833	0.447
1648	0.833	0.434
1759.5	0.833	0.421
1827	0.792	0.421
1906	0.792	0.408
1959.5	0.792	0.395
2077	0.75	0.395
2194	0.75	0.382
2211	0.75	0.368
2281	0.75	0.355
2354.5	0.75	0.342

2364	0.75	0.329
2375	0.708	0.329
2387	0.708	0.316
2397	0.708	0.303
2438.5	0.708	0.289
2476.5	0.667	0.289
2490.5	0.667	0.276
2517	0.625	0.276
2583	0.625	0.263
2655.5	0.625	0.25
2735.5	0.583	0.25
2798.5	0.583	0.237
2814.5	0.542	0.237
2839.5	0.542	0.224
2859.5	0.542	0.211
2934	0.5	0.211
3047.5	0.458	0.211
3103	0.458	0.197
3219	0.458	0.184
3442.5	0.458	0.171
3617	0.458	0.158
3895.5	0.417	0.158
4328.5	0.417	0.145
4581.5	0.417	0.132
4628.5	0.417	0.118
4939.5	0.375	0.118
5263	0.333	0.118
5332.5	0.333	0.105
5571.5	0.333	0.092
5985	0.333	0.079
6360.5	0.333	0.066
6574.5	0.333	0.053
6760.5	0.333	0.039
6897	0.333	0.026
7437.5	0.292	0.026
7991	0.292	0.013
8207	0.25	0.013
10351	0.208	0.013
13917.5	0.208	0
16600	0.167	0
24548	0.125	0
36895	0.083	0
128719	0.042	0
215071	0	0

Internal cross validation 4

DD(ug/L)	Sensitivity	1 - Specificity
8.8	1	1
159.9	1	0.987
345	1	0.974
391.5	1	0.961
405	1	0.947
408	1	0.934
434	1	0.921
459.5	1	0.908
479.5	1	0.895
500	1	0.882
560.5	1	0.868
662	1	0.855
714.5	1	0.842
733	1	0.829
744	1	0.803
748	1	0.789
759	1	0.776
772	1	0.763
797	1	0.75
821.5	0.958	0.75
826.5	0.958	0.737
843	0.958	0.724
858.5	0.958	0.711
876.5	0.958	0.697
903	0.958	0.684
944.5	0.958	0.671
988	0.958	0.658
1024	0.958	0.645
1065	0.958	0.632
1103.5	0.958	0.618
1128	0.958	0.605
1140.5	0.958	0.592
1166	0.958	0.579
1185.5	0.958	0.566
1190	0.958	0.553
1201.5	0.958	0.539
1213.5	0.958	0.526
1231.5	0.958	0.513
1255.5	0.958	0.5
1282.5	0.958	0.487
1320.5	0.958	0.474

1348	0.917	0.474
1357.5	0.917	0.461
1368.5	0.875	0.461
1383.5	0.875	0.447
1393	0.875	0.434
1405	0.875	0.421
1465	0.875	0.408
1542	0.875	0.395
1677.5	0.875	0.382
1868	0.833	0.382
1959.5	0.833	0.368
2070.5	0.792	0.368
2175.5	0.792	0.355
2194	0.792	0.342
2211	0.792	0.329
2281	0.792	0.316
2354.5	0.792	0.303
2374	0.792	0.289
2395	0.792	0.276
2443	0.792	0.263
2507.5	0.792	0.25
2606.5	0.792	0.237
2735.5	0.75	0.237
2798.5	0.75	0.224
2817.5	0.708	0.224
2842.5	0.667	0.224
2859.5	0.667	0.211
2910.5	0.625	0.211
2980.5	0.625	0.197
3027	0.583	0.197
3070.5	0.542	0.197
3091.5	0.542	0.184
3103.5	0.5	0.184
3219	0.5	0.171
3379	0.5	0.158
3498.5	0.458	0.158
3599	0.458	0.145
3654	0.458	0.132
3796.5	0.417	0.132
4020	0.375	0.132
4328.5	0.375	0.118
4581.5	0.375	0.105
4628.5	0.375	0.092
5194.5	0.333	0.092

5966	0.333	0.079
6341.5	0.333	0.066
6574.5	0.333	0.053
6760.5	0.333	0.039
6897	0.333	0.026
7467.5	0.292	0.026
8811	0.25	0.026
9751	0.25	0.013
12713.5	0.208	0.013
16600	0.167	0.013
18137.5	0.125	0.013
25011.5	0.125	0
36895	0.083	0
128719	0.042	0
215071	0	0

Internal cross validation 5

DD(ug/L)	Sensitivity	1 - Specificity
8.8	1	1
159.9	1	0.987
318.5	1	0.974
339.5	1	0.961
366	1	0.947
391.5	1	0.934
405	1	0.921
408	1	0.908
428.5	1	0.895
453.5	1	0.882
459.5	1	0.868
479.5	1	0.855
500	1	0.842
501.5	1	0.829
560.5	1	0.816
672	1	0.803
733	1	0.789
744	1	0.776
748	1	0.763
759	1	0.75
772	1	0.737
799.5	1	0.724
826.5	1	0.711
843	1	0.697
858.5	1	0.684
886.5	1	0.671

934	1	0.658
965.5	1	0.645
988	1	0.632
1004.5	1	0.618
1045.5	0.958	0.618
1103.5	0.958	0.605
1128	0.958	0.592
1140.5	0.958	0.579
1169.5	0.958	0.566
1200.5	0.958	0.553
1213.5	0.958	0.539
1223	0.958	0.526
1266.5	0.958	0.513
1320.5	0.958	0.5
1348.5	0.917	0.5
1368.5	0.875	0.5
1383.5	0.875	0.487
1393	0.875	0.474
1399	0.875	0.461
1406	0.875	0.447
1463.5	0.875	0.434
1622.5	0.875	0.421
1759.5	0.875	0.408
1827	0.833	0.408
1906	0.833	0.395
1959.5	0.833	0.382
2070.5	0.792	0.382
2175.5	0.792	0.368
2194	0.792	0.355
2211	0.792	0.342
2289.5	0.792	0.329
2364	0.792	0.316
2377	0.75	0.316
2430.5	0.75	0.303
2476.5	0.708	0.303
2490.5	0.708	0.289
2566	0.667	0.289
2712	0.667	0.276
2798.5	0.667	0.263
2814.5	0.625	0.263
2827	0.625	0.25
2842.5	0.583	0.25
2859.5	0.583	0.237
2910.5	0.542	0.237

3003.5	0.542	0.224
3070.5	0.5	0.224
3091.5	0.5	0.211
3103.5	0.458	0.211
3275	0.458	0.197
3498.5	0.417	0.197
3599	0.417	0.184
3654	0.417	0.171
3796.5	0.375	0.171
4020	0.333	0.171
4375.5	0.333	0.158
4939.5	0.292	0.158
5263	0.25	0.158
5332.5	0.25	0.145
5571.5	0.25	0.132
5966	0.25	0.118
6194	0.25	0.105
6360.5	0.25	0.092
6574.5	0.25	0.079
6760.5	0.25	0.066
7420.5	0.25	0.053
8177	0.25	0.039
8997	0.208	0.039
9751	0.208	0.026
11105	0.167	0.026
14991.5	0.167	0.013
18137.5	0.125	0.013
25011.5	0.125	0
36895	0.083	0
128719	0.042	0
