# Supplementary figures and images for: Predictive value of D-dimer and analysis of risk factors in pregnant women with suspected pulmonary embolism after cesarean section
Source: BMC Pulm Med. 2021 Dec 1;21:391. doi: 10.1186/s12890-021-01757-3 (PMC8638256; doi:10.1186/s12890-021-01757-3)

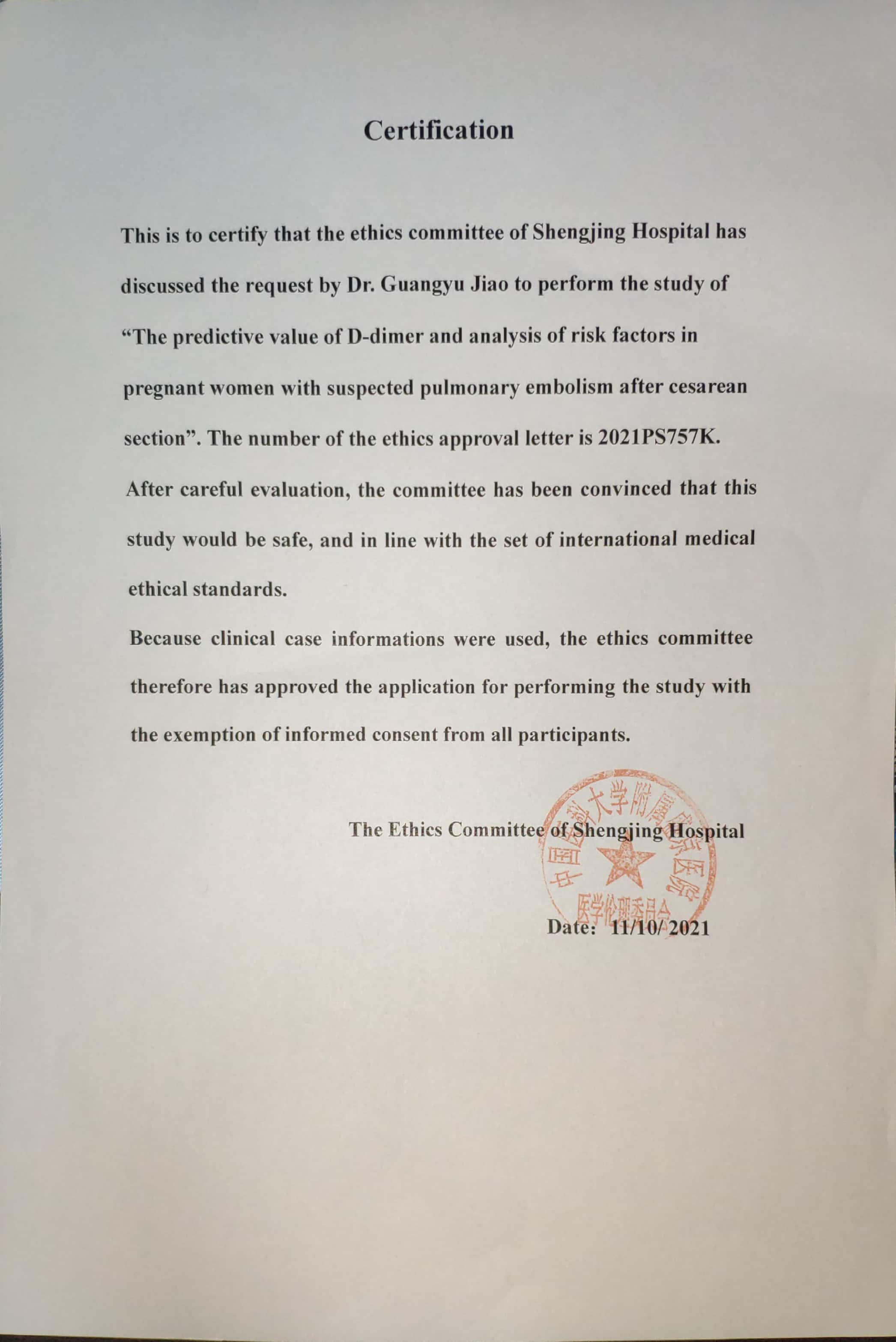

Supplement: Supplementary file 3 — Additional file 3. Ethical approval documents. [file 12890_2021_1757_MOESM3_ESM.jpg]
